# Simultaneous Determination of Refractive Index and Thickness of Submicron Optical Polymer Films from Transmission Spectra

**DOI:** 10.3390/polym13152545

**Published:** 2021-07-31

**Authors:** Víctor Bonal, José A. Quintana, José M. Villalvilla, Rafael Muñoz-Mármol, Jose C. Mira-Martínez, Pedro G. Boj, María E. Cruz, Yolanda Castro, María A. Díaz-García

**Affiliations:** 1 Departamento de Física Aplicada and Instituto Universitario de Materiales de Alicante, Universidad de Alicante, 03080 Alicante, Spain; victor.bonal@ua.es (V.B.); jmvs@ua.es (J.M.V.); rafa.marmol@ua.es (R.M.-M.); jcmm24@alu.ua.es (J.C.M.-M.); 2 Departamento de Óptica, Farmacología y Anatomía and Instituto Universitario de Materiales de Alicante, Universidad de Alicante, 03080 Alicante, Spain; ja.quintana@ua.es (J.A.Q.); p.boj@ua.es (P.G.B.); 3 Instituto de Cerámica y Vidrio (CSIC), Campus de Cantoblanco, 28049 Madrid, Spain; mariaeugenia@icv.csic.es (M.E.C.); castro@icv.csic.es (Y.C.)

**Keywords:** polymeric films, optical characterization, transmission spectra, dye-sensitized polymers

## Abstract

High-transparency polymers, called optical polymers (OPs), are used in many thin-film devices, for which the knowledge of film thickness (*h*) and refractive index (*n*) is generally required. Spectrophotometry is a cost-effective, simple and fast non-destructive method often used to determine these parameters simultaneously, but its application is limited to films where *h* > 500 nm. Here, a simple spectrophotometric method is reported to obtain simultaneously the *n* and *h* of a sub-micron OP film (down to values of a few tenths of a nm) from its transmission spectrum. The method is valid for any OP where the *n* dispersion curve follows a two-coefficient Cauchy function and complies with a certain equation involving *n* at two different wavelengths. Remarkably, such an equation is determined through the analysis of *n* data for a wide set of commercial OPs, and its general validity is demonstrated. Films of various OPs (pristine or doped with fluorescent compounds), typically used in applications such as thin-film organic lasers, are prepared, and *n* and *h* are simultaneously determined with the proposed procedure. The success of the method is confirmed with variable-angle spectroscopic ellipsometry.

## 1. Introduction

High-transparency polymer thin films, either pristine or doped with some other material, are increasingly used in the fabrication of electronic and optical devices, motivated by their low cost, easy fabrication and mechanical flexibility. Examples of such devices are organic field-effect transistors [1], light-emitting diodes [2], solar cells [3], or thin-film organic lasers (TFOLs) [4]. These highly transparent polymers, called optical polymers (OP) or organic glasses (to be distinguished from inorganic optical glasses), show transmittance values above 85% in a wide part of the visible and near-infrared (NIR) spectral region. In order to use these OP films in a given device, the refractive index, *n*, and the thickness, *h*, are very important parameters for optical and morphological characterization. Among the non-destructive methods described in the literature to measure *n* and *h*, ellipsometry and spectrophotometry are widely used. Ellipsometry is useful when high precision is required (four decimal places in *n* and accuracy within less than 1 nm in *h*), even when dealing with films of a few nanometers [5]. Spectrophotometry has lower accuracy, but it is cost-effective and the analysis is simpler and requires less time [6].

The standard spectrophotometric method developed by Swanepoel [7,8] is based on constructing the envelopes around the interference maxima and minima that appear in the transmission spectrum. However, the number of interference extremes decreases with the value of *h,* so accuracy decreases and the method becomes inapplicable when *h* < 500 nm [9]. Recently, our previous studies have proposed a spectrophotometric method to determine *h* down to ~50 nm, based on the comparison, in the transparent spectral window, between its experimental transmission spectrum at normal incidence and that obtained by simulation [10]. Even though this procedure enables us to measure very thin films, its application requires knowledge of the material refractive index dispersion, *n* (*λ*), or its determination when it is unknown. This complicates the overall procedure because it requires either the preparation of an additional film of the same material, but with *h* > 1 μm, to determine *n* by the standard Swanepoel’s method, or to use alternative techniques, such as Abelès, to measure *n* independently, for at least two different wavelengths [10].

Precise *n* data for OPs in the visible and especially in the NIR spectra are relatively sparse [11,12]. Besides, the refractive index of polymers may vary from one producer to another, since it is influenced by many fabrication factors, such as the degree of polymerization and the processing temperature [13]. In order to obtain the *n* (*λ*) for a given material, a general procedure is to measure *n* at several wavelengths and then select a proper function, such as the Cauchy equation, to adjust the data [14]. In fact, this could be further simplified to limit the number of required experimental wavelengths to only one value (the refractive index, *n_λ_*_0_, at a reference wavelength, *λ*_0_), if it is possible to find an equation that relates *n_λ_*_0_ to the index at another wavelength, *n_λ_*_1_. This is equivalent to finding the dependence on *n_λ_*_0_ of the strength of the dispersion, quantified by Δ*n* = *n_λ_*_1_ − *n_λ_*_0_.

Here, a spectrophotometric method to obtain simultaneously the *n* and *h* of sub-micron OP films (values down to a few tenths of a nm) from a single measurement of its transmission spectrum is reported. This method is very simple, is widely available as it only requires a spectrophotometer, and provides significant savings in work and time, compared to the previously reported spectrophotometric methods for such a purpose. Upon the analysis of precise experimental *n* data of 16 different OPs reported by Sultanova et al. [12], an equation that relates with a good precision *n_λ_*_0_ to Δ*n* has been found, demonstrating its validity for most OPs. Then, the simultaneous determination of *h* and *n* for a given OP film can be reduced to a calculation problem with two variables, *n_λ_*_0_ and *h*, which can be solved through the application of a simple program where the experimental and calculated transmittance spectra are compared. Since *n_λ_*_0_ is one of the unknowns, this procedure would also be applicable when *n* varies with *h* due to a change in its physical properties, such as, for example, when *h* < 100 nm [10,15]. The validity of the proposed spectrophotometric method is then demonstrated for OPs of various types: commercially available polymers, natural biopolymers and polymers with a dispersed amount of an organic active compound. As commercially available polymers, polystyrene (PS) and polymethyl methacrylate (PMMA) have been chosen, as they are often used in plastic optoelectronics applications. The natural biopolymer used is gelatin, the refractive index of which depends on the amino-acid composition, and its interest is based on the use of its dichromated version (dichromated gelatin (DCG)) to fabricate distributed feedback polymeric resonators by holographic methods for TFOLs [4,16,17]. Finally, as dye-doped polymers, we opted for PS films with a dispersed amount of an organic active compound, intended to provide a certain functionality, such as light emission, for their use as active layers of TFOLs. For this purpose, three active organic compounds have been used: triphenyl-diamine (TPD) [18], perylene orange (PDI-O) [19], and a nanographene derivative (FZ3) [20], which emit in the blue, yellow and NIR spectral regions, respectively. In the case of TPD, the dye-doping rate was varied in a wide range to analyze the effect of this on the refractive index. To confirm the results and to estimate the remaining error of the proposed spectrophotometric method, variable angle spectroscopic ellipsometry (VASE) is employed.

## 2. Materials and Methods

### 2.1. Refractive Index Dispersion Analysis of OPs

Precise *n* data (to four decimal places), measured with a Pulfrich refractometer at 9 different wavelengths (between 435 and 1055 nm) by Sultanova et al. [12] for a set of 16 different OPs, have been considered. They include commonly used OPs, such as PMMA, PS, polycarbonate (PC) and the styrene–acrylonitrile copolymer (SAN); different trademarks: CTE-Richardson, NAS-21, S-low-styrene, Optorez 1330, Zeonex E48R and Bayer; finally, other OPs such as cellulose, polyacrylate, styrene, acrylic, EBM copolyester and S-copolyester, produced by the USA Eastman Chemical Company (ECC, Kingsport, TN, USA).

**Figure A1:**
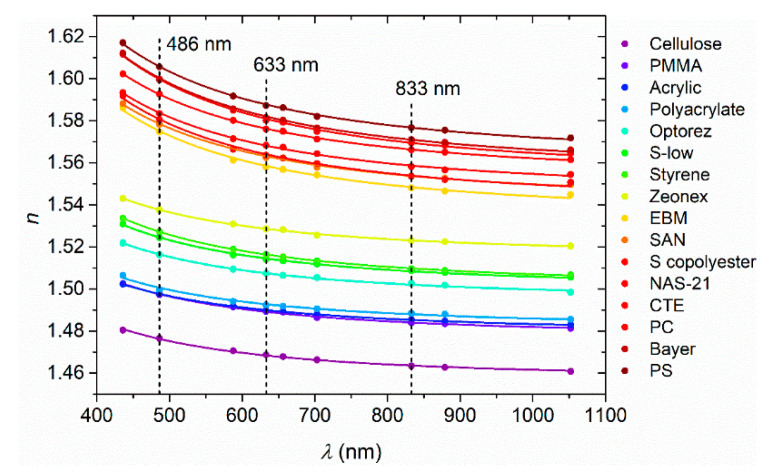
Dispersion curves for 16 OPs (see legend), obtained by fitting experimental *n* data (symbols) extracted from reference [12], with Cauchy equation (equation 1) with two coefficients.

Since OPs are transparent and isotropic materials, these experimental *n* data can be fitted in the spectral region of normal dispersion (Figure A1) by using Cauchy’s equation:

*n*(*λ*) = A + B/*λ*^2^ + C/*λ*^4^ + D/*λ*^6^ + ··· (1)

where A, B, C, D, etc. are the Cauchy’s coefficients. A possible procedure to analyze the *n* dispersive behavior of OPs without the need of a large set of experimental data, but with just three experimental data points, consists of using the so-called Abbe number, *υ*_D_:

*υ*_D_ = (*n*_D_ − 1)/(*n*_F_ − *n*_C_) (2)

where *n*_D_, taken as the reference, is the index at the yellow D-line wavelength of the sodium lamp (589.29 nm), and *n*_F_ − *n*_C_ is the difference between the indices at the blue F-line and at the red C-line wavelengths of the hydrogen lamp, at 486.13 and 656.28 nm, respectively. Therefore, to obtain the Abbe number, it is necessary to know *n* at three different wavelengths. However, this method is only useful in the visible spectrum (see Supplementary Materials Figure S1).

**Figure 1:**
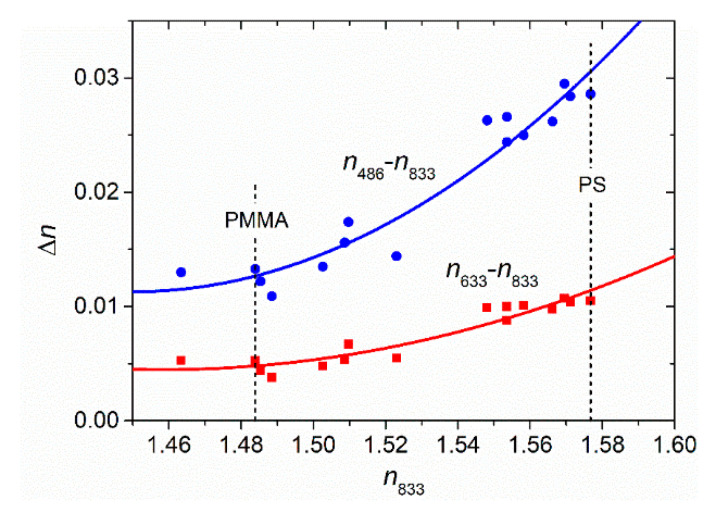
Partial dispersions *n*_633_ − *n*_833_ (red squares) and *n*_486_ − *n*_833_ (blue circles) as a function of *n*_833_, for a series of OPs (*n* data taken from ref. [12]) fitted (solid lines) with our proposed second-order polynomial model (Equation (3) for *n*_633_ − *n*_833_ and Equation (4) for *n*_486_ − *n*_833_). The R^2^ parameters for the fits are 0.89 and 0.92, respectively).

In order to simplify the process to obtain the *n* dispersion curve of a given material by using the lowest possible number of parameters while maintaining a good precision, a procedure consisting of finding the strength of a partial dispersion, Δ*n* = *n_λ_*_1_ − *n_λ_*_0_, as a function of *n* at a reference wavelength, *n_λ_*_0_, has been considered. Remarkably, if an equation relating Δ*n* with *n_λ_*_0_ is found, it would be possible to obtain the *n* dispersion curve knowing only one *n* data point, using Equation (1) with two coefficients (A and B). Besides, this equation could be used for the purpose of obtaining *h* and *n* from the transmission spectrum. To extend the validity of the method to the NIR, *λ*_0_ = 833 nm was selected as the reference wavelength. For the other wavelength, *λ*_1_, two spectral lines used by Sultanova, 633 and 486 nm, were examined. Then, by selecting the experimental *n* data for the whole set of OPs at these three wavelengths, the partial dispersion Δ*n*, i.e., *n*_633_ − *n*_833_ (or *n*_486_ − *n*_833_) was represented as a function of *n*_833_ (see Figure 1). After trying different possible functions to fit the data (considering that polymers with higher refractive index show larger dispersion (see Figure A1), we found that a second-order polynomial function provided the best results (full lines in Figure 1). Assuming *n*_833_ is known, the mean of absolute deviations from the real values, ±0.0007 and ±0.0014 at *n*_633_ and *n*_486_, respectively, are relatively small.

The relationships between *n_λ_*_0_ and Δ*n*, obtained as a result of these fits, are:

*n*_633_ − *n*_833_ = 1.505574 − 1.44177 *n*_833_ + 0.49434 *n*_833_^2^ (3)

*n*_486_ − *n*_833_ = 2.53646 − 3.48287 *n*_833_ + 1.20095 *n*_833_^2^ (4)

**Figure 2:**
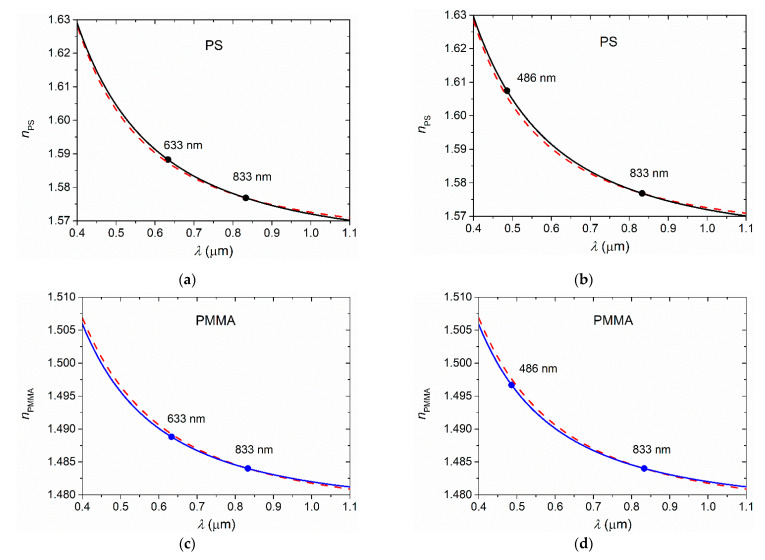
Dispersion curves for polymers PS (**a**,**b**; black solid lines) and PMMA (**c**,**d**; blue solid lines), obtained with the two-coefficient Cauchy model, assuming known *n*_833_, and with *n*_633_ (**a**,**c**) or *n*_486_ (**b**,**d**) calculated with Equations (3) or (4), respectively. Full circles correspond to known *n* values (*n*_833_) and calculated *n* ones (*n*_633_ or *n*_486_). To illustrate the validity of the model the precise dispersion curves of these two polymers (taken from Figure A1) are also included (red dashed lines).

The validity of Equations (3) and (4), combined with the two-coefficient Cauchy’s equation to obtain the dispersion curve *n* (*λ*), using *n*_833_ as the only known amount, has been verified for two of the principal polymers of the considered set, PMMA (*n*_833_ = 1.4839) and PS (*n*_833_ = 1.5767), the data points of which are labeled in Figure 1. The dispersion curves obtained with this method for these two polymers are very precise (Figure 2): absolute deviations from the real values for PMMA are 0.0004 and 0.0009 at *n*_633_ and *n*_486_, respectively, somewhat smaller than the mean of absolute deviations, and for PS are 0.0009 and 0.0018 at *n*_633_ and *n*_486_, respectively, somewhat larger than the mean of absolute deviations. We have also considered the Zeonex E48R polymer that, from the Sultanova set, is the one that deviates the most from the fitted curve. In this case, the differences between the calculated index values and the real ones are ~0.001 for *n*_633_ and ~0.003 for *n*_486_. It can also be seen that the dispersion has similar precision to that obtained when all experimental data fit with Equation (1), as in Figure A1.

Refractive index data of some common transparent commercial polymers not considered by Sultanova, which also approximately satisfy Equations (3) and (4), are presented in Table 1: poly-*N*-Isopropylacrylamide (PNIPAM) [21], polyvinylpyrrolidone (PVP) [22], polyvinylchloride (PVC) [23], polyethylene terephthalate (PET) [23], polyester from ECC [24], and ethylene vinyl acetate (EVA) from Bridgestone Evasky S87 [25]. The gelatin biopolymer Rousselot 1313, used for many years in our laboratory (measurements by VASE), and the synthetic polymer polyvinyl alcohol (PVA) [26], both water-soluble, have also been included in Table 1. The blending of polymers has also been considered because it is a simple way to modify the physical properties of the blend material, including the refractive index [27]. In these cases, special care must be taken to maintain the high transparency inherent to OPs. Depending on the miscibility, the blend morphology ranges from a single-phase system to two or multiphase systems. In binary blending, the transparency of the mixture decreases as the content of the disperse phase and the difference between the refractive indices of both polymers increase [28]. However, it has been shown that the extinction coefficient is maintained at a low level (<10^−5^) by adding a small proportion of PC to the PMMA, with indices that are appreciably different [29]. Data corresponding to the blend PMMA/20%PC [30] are included in Table 1. Finally, the bisphenol A-based poly (phosphonate) (PC1) [31], with a higher refractive index than those considered by Sultanova, has also been included in Table 1. It can be seen that the index values obtained by using Equations (3) or (4) have a similar precision to that obtained with the set considered by Sultanova.

Finally, it should be noted that not all OPs satisfy Equations (3) and (4). In fact, transparent polymers, such as poly (thioether sulfones) [32], and sulfur-containing poly (meth)acrylates [33], which have been synthesized for some special applications, present a high index maintaining a relatively small dispersion (high Abbe number).

### 2.2. Method for Simultaneous Determination of n and h in OP Thin Films

An OP thin film coated on a thick transparent substrate of index *s* has been considered. Calculations are based on the comparison, in the transparent spectral window, between the experimental transmission spectrum at normal incidence and that obtained by simulation. Since the comparison takes place in the transparent window, the simulation of the spectrum can be performed by using a relatively simple equation of transmittance [7]:

T = A/(B − C cos φ + D)(5)

where *A* = 16*n*^2^*s*, *B* = (*n* + 1)^3^ (*n* + *s*^2^), *C* = 2 (*n*^2^ − 1)^3^ (*n*^2^ − *s*^2^), *D* = (*n −* 1)^3^ (*n − s*^2^) and *φ = 4πnh/λ*.

This equation describes the interference between the directly transmitted light and that transmitted after two internal reflections in the film. Here, OP films are spin-coated on 1-mm thick transparent fused silica (FS) substrates. Thus, the substrate, which has a thickness that is several orders of magnitude larger than *h*, does not affect the interference pattern. The equation corresponding to the interference-free transmission spectrum of a thick transparent uncoated substrate:

*T*_FS_*=* 2s/(s^2^ + 1) (6)

will also be considered below.

As explained in the previous section, the knowledge of *n* (*λ*) between 400 and 1100 nm for an OP can be reduced to the knowledge of *n*_833_ and, as a consequence, according to Equation (5), the spectrum in the transparent window can be calculated using only two parameters, *n*_833_ and *h*. The comparison between the spectrum measured in the spectrophotometer, *T*_exp_, and those generated by Equation (5), *T*_calc_, can be carried out using a simple Mathematica program (Wolfram Research, Inc.) in an iterative process until the difference between *T*_exp_ and *T*_calc_ is minimal. In the program, the spectrum against air provided by the spectrophotometer, and the dispersion curve of the FS, *s* (*λ*), represented with the Malitson equation [34], are the input data. For each value of the variable *n*_833_, the program constructs the dispersion curve, *n* (*λ*), using Equation (1) with two coefficients and Equation (3) or (4). In this work, calculations have been made using Equation (3) because it is useful for the three types of OPs studied. Then, Equation (5) is tabulated for each pair of values (*n*_833_, *h*), and compared in the transparent window of the material with the experimental data. The difference between both spectra is evaluated, using as merit function the sum of the absolute values of the deviations, R:

R *=* Σ*_i_* |*T*_exp_(*λ*_i_) − *T*_calc_(*λ*_i_)| (7)

**Figure A2:**
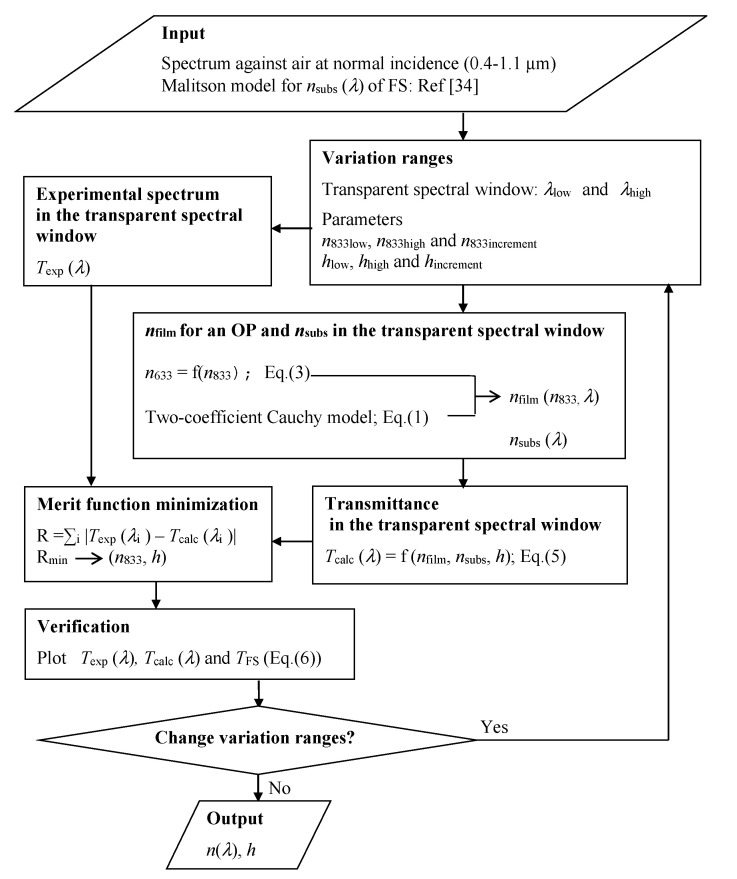
The flowchart of the program for calculating the refractive index *n* and thickness *h* of optical polymer (OP) films from transmission spectra in the visible and NIR. The program can also be applied to doped OP films since the accuracy of the method is maintained, even if the transparent spectral window is as small as 200 nm. The numbers of equations and references correspond to those in the main text. The computation time depends on the range and accuracy of the variables considered. However, with a little practice, it can be reduced to a few seconds. It is useful to carry out a first test with low accuracy, to obtain approximate values of *n* and *h*. Thus, in a second test, the range can be reduced and the accuracy can be increased.

The minimum value of R leads to the index *n*_833_ and the thickness of the film *h*. Finally, *T*_exp_ and *T*_calc_ are plotted to confirm the coincidence. This final step allows us to find out cases, such as those cited at the end of the previous section, in which the method cannot be applied because the equation that relates the values (*n*_λ1_ − *n*_λ0_) and *n*_λ0_ is not fulfilled. A flowchart of the program can be seen in Figure A2

### 2.3. Film Preparation and Optical Measurements

For the verification of the method, films of various types of materials were prepared: (i) commercial polymers used as received, PS (Merck, Mw = 35,000 g/mol) and PMMA (Merck, Mw = 350,000 g/mol), of high and low *n* values, respectively; (ii) a biopolymer gelatin (Rousselot 1313), with a medium *n* value; (iii) PS films doped with organic functional compounds, TPD (Merck, Kenilworth, NJ, USA), PDI-O (Phiton, Tittmoming, Germany) and nanographene FZ3 (provided by Prof. J. Wu; synthesis reported in [20]).

OP films were prepared by spin-coating (30 s, 3000 rpm) over FS substrates (size 25 × 25 × 1 mm^3^; Creator Optics Inc.). The solvent was toluene in all cases, except for the gelatin, for which hot water (40 °C) was used. The concentration of the polymer in the solvent (this value is used to identify a given film) was adjusted to obtain films with different thicknesses. Films with *h* < 500 nm were prepared (although our method to determine *h* and *n* is valid for films up to several microns thick) because, in this regime, standard spectrophotometric methods are not applicable. Several films with *h* < 200 nm were prepared to find the limit for method applicability.

Transmission measurements on the films were performed in a double-beam spectrophotometer (Jasco V-650, Jasco, Tokyo, Japan) equipped with a photomultiplier tube detector (for wavelengths below 900 nm). It is important to note that OP transmittance variations with wavelengths between 400 and 1100 nm are often relatively low (for example, between 0.935–0.885, absorbance 0.029–0.053, for undoped thermoplastic polymers). Thus, it is convenient to use a spectrophotometer equipped with a high-sensitivity detector to record low noise spectra. Measurements were taken against air at normal incidence in the center of the sample over the area of the slit image of about 1 mm × 7 mm, between 400 and 900 nm, with light of a spectral bandwidth of 1 nm. Measurements at wavelengths higher than 900 nm (for example with FZ3) were made with a Jasco V-670 UV-VIS-NIR spectrophotometer (Jasco, Tokyo, Japan) to reduce the noise. VASE measurements, to further validate our method, were performed with an M-2000 U instrument (Abbott, IL, USA), using a film surface area of 4 × 5 mm^2^ in the center of the sample.

## 3. Results

In this section, the validity of the method for films of different OPs is demonstrated.

### 3.1. Commercial OPs

First, the transmission spectra for a set of PS films were measured. The percentage of polymer in the solvent was different (ranging between 0.9 and 8 wt %) aiming to obtain different thicknesses. Then, the analysis method previously described was used to obtain *n* at the reference wavelength (833 nm) and *h*. The results are collected in Table 2, where measurements made with VASE are also included for comparison purposes. It is seen that the method has high accuracy. Remarkably, the increase in the refractive index for very thin samples, shown in VASE measurements, is also found in measurements performed with the proposed spectrophotometric method. It should be noted that the value of the *n*_833_ measured by Sultanova for the bulk of a PS sample, 1.5767, should correspond to sufficiently thick films.

**Figure 3:**
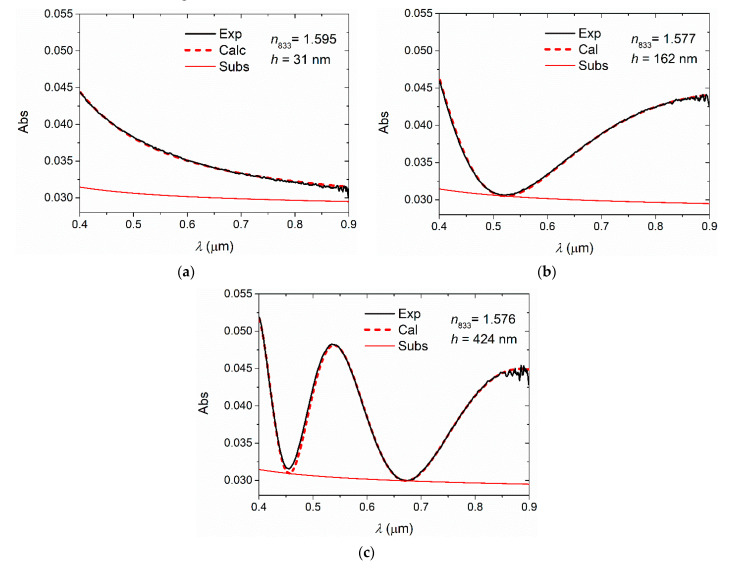
Experimental absorption spectra against air (black solid lines) and calculated interference patterns (red thick dashed lines) corresponding to PS films: (**a**) PS 0.9%, (**b**) PS 3.0%, and (**c**) PS 8.0%. The substrate spectrum Equation (6) has also been included (red solid lines).

Figure 3 shows experimental absorption spectra against air, and simulated interference patterns for three of the samples considered in Table 2. As usual in organic electronics, spectra are expressed using absorbance (Abs) instead of transmittance. The trace corresponding to the substrate, according to Equation (6), has also been included. The spectral curve should be tangent to this curve at the interference minima [7,10]. However, it is noted that most OPs present a small absorption at short wavelengths of the visible spectrum, leading to a decrease in transparency when *h* is large. Thus, the minima of the spectral curves are no longer tangential to the substrate curve for the shorter wavelengths (see Figure 3c). Therefore, it is suitable to apply the method in a window of wavelengths starting in 500 or 600 nm for *h* greater than about 250 nm. The reduced transparent spectral window can be a problem when analyzing very thin films because the trace is very simple and the error increases, as data can be fitted with multiple values of *n* and *h*. In this case, since the spectral trace for these *h* values is sufficiently complex, a transparent spectral window of about 200 nm is sufficient to compare the experimental and calculated data; the error remains small as data can only be fitted by a single pair of values of *n* and *h*. Experimental and calculated spectra corresponding to all samples in Table 2 are shown in Figure S2.

The method to determine *n* and *h* has also been used for films of two other commercial OPs with lower refractive indexes: PMMA and gelatin. Table 3 compares the *n* and *h* values of a set of PMMA films, obtained with the proposed method, to those measured more accurately by VASE. The value of the *n*_833_ reported by Sultanova for the bulk of a PMMA sample is 1.4839, slightly higher than that obtained by VASE. The results corresponding to a Rousselot 1313 gelatin film are also included. Experimental and calculated spectra corresponding to all samples in Table 3 are shown in Figure S3.

### 3.2. Dye-Doped OP

**Figure 4:**
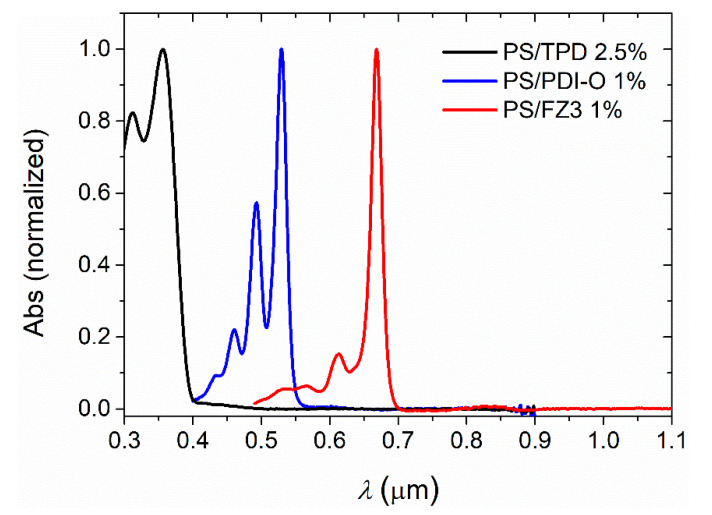
Absorption spectra of the organic dyes TPD, PDI-O and FZ3. Interference patterns, which can modify appreciably the wavelength and relative intensity of absorption peaks, have been subtracted to obtain true absorption spectra of the active materials.

The procedure for calculating *n* and *h* was also applied to films of an inert OP doped with an organic dye, such as those used to prepare the active layers of TFOLs. Even though these dyes increase the absorbance over a limited interval of wavelengths, calculations can be performed on the transparent part of the spectrum, where the proposed method is valid. The absorption spectra of the organic dyes considered in the present research, TPD and PDI-O, which emit in the blue and the yellow parts of the visible spectrum, respectively, and the nanographene FZ3, which emits in the near-IR, are shown in Figure 4. In the three cases, absorption takes place in a band of wavelengths shorter than those of the corresponding emission so that the transparent window is located at longer wavelengths. The method is applied in the range of 600–850 nm, using Equation (3) for samples with TPD and PDI-O. On the other hand, polymers doped with dyes such as FZ3, which are only transparent in the NIR spectral region (*λ* > 700 nm), require a new auxiliary equation. This equation was obtained by fitting the partial dispersion *n*_833_ − *n*_1052_ as a function of *n*_833_ for the Sultanova polymer set (Figure A1):

*n*_833_ − *n*_1052_ = 0.57101 − 0.76428 *n*_833_ + 0.25700 *n*_833_^2^ (8)

Measurements of *n* and *h* are presented in Table 4. The amount of dye that can be usefully introduced for PDI-O and FZ3-doped PS films is limited to low concentrations (1 wt %) due to photoluminescence quenching [19,20]. On the other hand, active layers of lasers based on TPD can be doped with a dye concentration that is considerably higher [18], so that the limitation of the proposed method to these materials could be studied. As expected, in this case, the refractive index increases proportionally to the dye concentration, according to the Arago–Biot equation [10]. The comparison with the data measured by VASE indicates that i), the accuracy of the method is maintained even if the transparent window is as small as 200 nm, and ii), the method can be applied to dye concentrations up to 30 wt %. Experimental and calculated spectra, corresponding to all samples in Table 4, are shown in Figure S4.

## 4. Discussion

**Figure 5:**
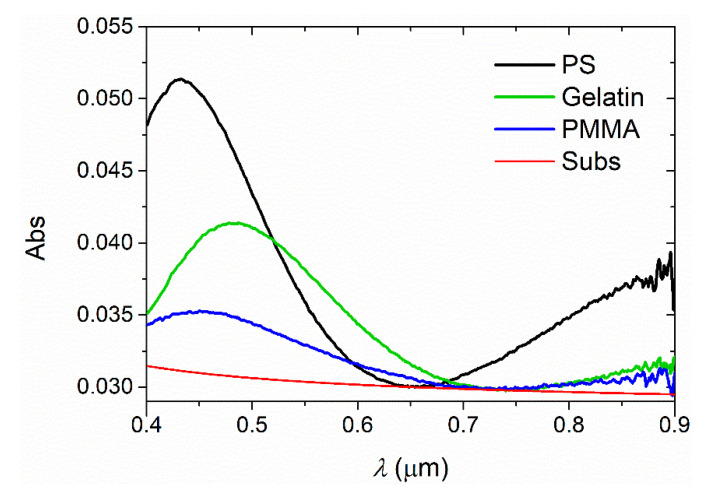
Experimental absorption spectra corresponding to PS (*n*_833_ = 1.575), gelatin (*n*_833_ = 1.526) and PMMA (*n*_833_ = 1.484) films with *h* in the range of 200–250 nm. The theoretical absorption spectrum calculated with Equation (6) has also been included (red solid line).

An important aspect to discuss is the accuracy of the method. We note that *n* values depend mainly on the difference (contrast) between the refractive index of the film (*n*) and the substrate (*s*), that is, (*n* − *s*), while *h* values depend mainly on the period of the interferences. This is illustrated qualitatively in Figure 5, which shows absorption spectra for selected films of PS, gelatin and PMMA, for which the (*n* − *s*) values are different. It is seen that a decrease in (*n* − *s*) leads to a decrease in the interference contrast (absorbance difference between maximum and minimum). As a consequence, when (*n* − *s*) decreases (in Figure 5, when moving from PS to gelatin, and to PMMA), the interference fringes are less defined; therefore, the accuracy also decreases. In fact, the substrate index *s*, here, that of FS (*n*_833_ = 1.453), is one theoretical limit of the proposed method. One way to increase the accuracy of the method would be to use a lower index substrate, such as polydimethylsiloxane (PDMS), the refractive index *n*_833_ of which is 1.40. However, this possibility first requires the development of a strategy to improve the compatibility of this material with nonpolar solvents [35]. Since the index of the substrate can be lower or higher than that of the polymer, another, simpler option that could be explored is to use a substrate, such as a dense flint glass, with *n* > 1.63.

**Figure 6:**
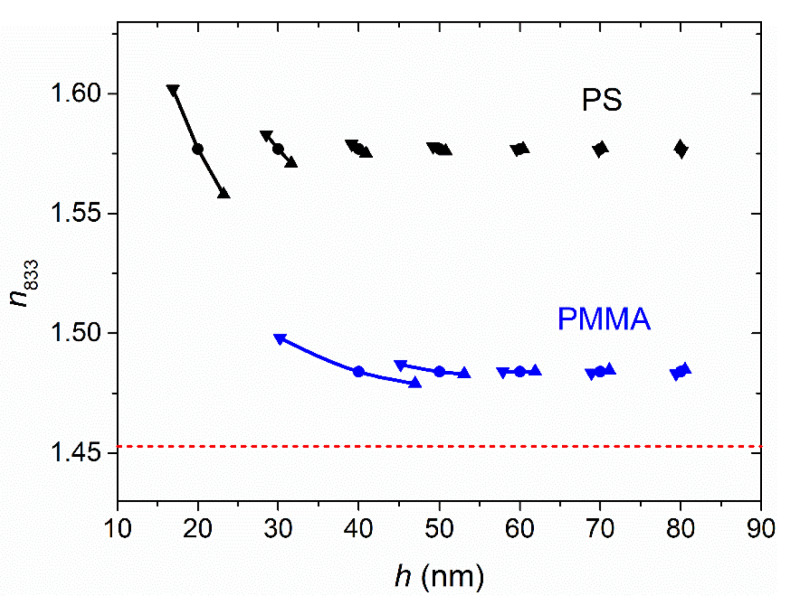
Changes in *n*_833_ and *h* of PS and PMMA films due to a systematic error of ±0.0001 in absorbance (triangles pointing up for data with error +0.0001 and triangles pointing down for data with error −0.0001). The substrate *n*_833_ has also been included (red dashed line).

It is also important to discuss the instrumental systematic errors associated with the use of the proposed method. Even though these errors can be reduced by regular calibration and maintenance, we found that the comparison of the spectrum of a clean uncoated substrate with the theoretical spectrum obtained from Equation (6) is a good test, in order to incorporate, if necessary, a correction factor in measurements. So, in our case, we have observed that residual systematic deviations of absorbance are in the order of ± 0.0001. Changes in *n* and *h* for absorbance increments of this order in the whole spectrum are shown in Figure 6. It can be seen that the errors of *n* and *h* increase rapidly for small thicknesses. Besides, the error of *h* increases faster than that of *n* in the case of PMMA, for which (*n* − *s*) is small. So, we determined that the lowest film thickness values that could be accurately measured (Δ*h* < 5 nm) were around 30 and 60 nm for PS and PMMA, respectively. Above these limits, the remaining errors were estimated through a comparison of the results with those obtained by VASE, which were taken as a reference. According to the results in Table 2 and Table 3, the means of absolute deviations in the *n*_833_ and *h* values are 0.003 and 2 nm for PS, and 0.006 and 4 nm for PMMA, respectively. Thus, in our experimental conditions, accuracy for PMMA films decreases by a factor of about 2 relative to PS films.

## 5. Conclusions

The proposed method for the simultaneous determination of *n* and *h* of OP materials represents a significant improvement over previously reported spectrophotometric procedures, particularly for measuring *h* < 500 nm, due to the savings in work and time involved. The novelty of the method is the finding of an optical general behavior of OPs, derived from the fact that higher refractive index polymers show larger dispersion. This allows us to obtain an equation relating Δ*n* with an index value *n*_λ0_, and, in the last term, to determine the *n* and *h* of an OP thin film by comparing the experimental with the calculated transmission spectrum. The method has also been extended to dye-doped OP films, making the comparison in a transparent part of the spectrum where the proposed procedure is valid. This important application is possible because the accuracy is maintained, even if the transparent window is as small as 200 nm.

Precise dispersion curves between 400 and 1100 nm for the transparent OP materials can be obtained by taking *n*_833_ as the reference index, and the index variation *n*_633_ − *n*_833_ as the magnitude of the dispersion. For most OP materials, *n*_633_ can also be related with *n*_833_ by a second-order polynomial equation, so it is enough to determine *n*_833_ to obtain the dispersion curve by using the two-coefficient Cauchy’s formula. Thus, the spectrum of an OP film can be expressed by an equation with two unknowns, the index *n*_833_ and the thickness *h*, which values can be simultaneously determined by comparison with the experimental spectrum.

Accuracy decreases as the difference between the index of the film and that of the substrate decreases. Considering that the polymers PS and PMMA have indices around 1.59 and 1.49, respectively, and the FS substrate has an index around 1.45, the minimum measurable thickness is approximately 30 nm for PS films and 60 nm for PMMA films. Above these minimum thicknesses, errors of index and thickness were estimated to be 0.003 and 2 nm for PS, and 0.006 and 4 nm for PMMA.

## Figures and Tables

**Table 1:** Verification of the validity of our model to obtain *n*_633_ and *n*_486_ with equations (3) and (4), respectively, using experimental *n*_833_ data taken from differences sources (indicated references). The calculated values are compared to the corresponding experimental ones reported in the same sources.

Polymer	Ref.	*n*_833_	*n*_633_	*n*_633_ (Equation(3))	*n*_486_	*n*_486_ (Equation(4))
PVA	[26]	1.470	1.477	1.475	1.488	1.482
EVASKY S87	[25]	1.488	1.492	1.493	1.500	1.501
Polyester (ECC)	[24]	1.491	1.496	1.496	1.505	1.504
PNIPAM	[21]	1.497	1.501	1.502	1.510	1.511
PVP	[22]	1.520	1.525	1.526	1.536	1.537
PMMA/PC 20%	[30]	1.520	1.524	1.526	1.533	1.537
Gelatin 8%	VASE	1.535	1.541	1.542	1.552	1.555
PVC	[23]	1.534	1.540	1.541	1.550	1.554
PET	[23]	1.556	1.565	1.565	1.584	1.581
PC1	[31]	1.595	1.606	1.608	1.627	1.631

**Table 2:** Comparison of *n* and *h* of PS films, obtained with the proposed spectrophotometric method (SP) and with VASE.

Sample	*n*_833_		*h* (nm)	
Polymer % in Solvent (Spin-Coat)	SP	VASE	SP	VASE
PS 0.9%	1.595	1.598	31	31.76
PS 1.2%	1.579	1.585	48	48.62
PS 1.9%	1.576	1.581	82	79.44
PS 3.0%	1.577	1.578	162	161.6
PS 4.6%	1.577	1.575	204	210.5
PS 6.7%	1.577	1.575	335	335.9
PS 8.0%	1.576	1.574	424	427.0

**Table 3:** Comparison of *n* and *h* of PMMA and gelatin films, obtained with the proposed spectrophotometric method (SP) and with VASE.

Sample	*n*_833_		*h* (nm)	
Polymer % in Solvent (Spin-Coat)	SP	VASE	SP	VASE
PMMA 2.8%	1.492	1.480	64	67.15
PMMA 3.2%	1.486	1.478	88	89.72
PMMA 3.5%	1.484	1.479	101	105.0
PMMA 5.0%	1.484	1.480	234	236.7
PMMA 7.0%	1.483	1.479	339	344.3
PMMA 10%	1.484	1.480	529	532.0
Gelatin 4.0%	1.528	1.526	241	236.0

**Table 4:** Comparison of *n* and *h* of dye-doped PS films, obtained with the proposed spectrophotometric method (SP) and with VASE. The fitting for PDI-O and TPD was made in the range of 600–850 nm, and FZ3 was fitted in two ranges: 750–850 nm and 750–1500 nm (using data from the Jasco V-670 UV-VIS-NIR spectrophotometer).

Sample	*n*_833_		*h* (nm)	
Polymer/Dye (Dye wt % in Polymer)	SP	VASE	SP	VASE
PS/PDI-O (1%)	1.580	1.581	522	521.9
PS/TPD (2.5%)	1.581	1.581	374	378.9
PS/TPD (5%)	1.580	1.583	352	349.7
PS/TPD (15%)	1.592	1.594	347	344.0
PS/TPD (30%)	1.607	1.609	324	316.9
PS/FZ3 (1%)	1.575	1.579	643	647.9
PS/FZ3 (1%) V-670	1.579	1.579	647	647.9

## Data Availability

The data supporting the findings of this manuscript are available from the corresponding authors upon reasonable request.

## Supplementary Materials

The following are available online at https://www.mdpi.com/article/10.3390/polym13152545/s1. Figure S1: Abbe number as a function of *n*_D_ for the set of OPs considered in Figure 1. Figure S2: Experimental absorption spectra against air and simulated interference patterns corresponding to PS films in Table 2 of the main text. Figure S3: Experimental absorption spectra against air, and simulated interference patterns corresponding to samples in Table 3 of the main text. Figure S4: Experimental absorption spectra against air, and simulated interference patterns corresponding to doped PS films in Table 4 of the main text.

## Appendix A

The proposed method for simultaneous determination of *n* and *h* of OP materials is based on the fact that in this group of materials, higher refractive index polymers show larger dispersion, so that an equation relating Δ*n* with an index value *n*_λ0_, which is taken as a reference, can be obtained. Figure A1 shows dispersion curves for a set of 16 different polymers which show such a behavior.

The flowchart of the program for calculating the refractive index *n* and thickness *h* of optical polymer (OP) films from transmission spectra in the visible and NIR is shown in Figure A2.
