# Supplementary material for: Simultaneous Determination of Refractive Index and Thickness of Submicron Optical Polymer Films from Transmission Spectra
Source: Polymers (Basel). 2021 Jul 31;13(15):2545. doi: 10.3390/polym13152545 (PMC8348323; doi:10.3390/polym13152545)
Supplement: Supplementary file 1 [file polymers-13-02545-s001.zip › polymers-1312341-supplementary.pdf]

Simultaneous Determination of Refractive Index and Thickness of Submicron Optical Polymer Films from Transmission Spectra

Víctor Bonal [1], José A. Quintana [2], José M. Villalvilla [1], Rafael Muñoz-Mármol [1], José C. Mira-Martínez [1], Pedro G. Boj [2], María E. Cruz [3], Yolanda Castro [3] and María A. Díaz-García [1,*]

[1] Departamento de Física Aplicada and Instituto Universitario de Materiales de Alicante, Universidad de Alicante, 03080 Alicante, Spain; victor.bonal@ua.es (V.B.); jmvs@ua.es (J.M.V.); rafa.marmol@ua.es (R.M.-M.); jcmm24@alu.ua.es (J.C.M.-M.)
[2] Departamento de Óptica, Farmacología y Anatomía and Instituto Universitario de Materiales de Alicante, Universidad de Alicante, 03080 Alicante, Spain; ja.quintana@ua.es (J.A.Q.); p.boj@ua.es (P.G.B.)
[3] Instituto de Cerámica y Vidrio (CSIC), Campus de Cantoblanco, 28049 Madrid, Spain; mariaeugenia@icv.csic.es (M.E.C.); castro@icv.csic.es (Y.C.)
* Correspondence: maria.diaz@ua.es

Keywords: polymeric films; optical characterization; transmission spectra; dye-sensitized polymers

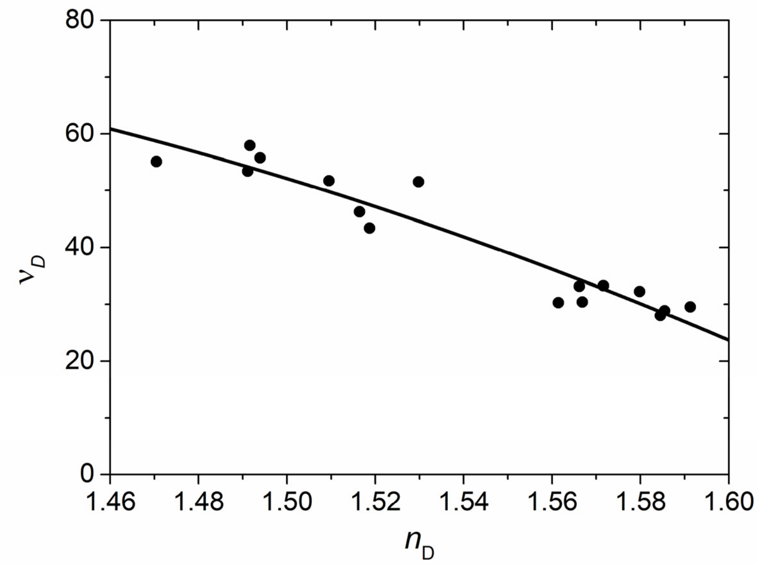

Figure S1. Abbe number as a function of n_D for the set of OPs considered in Fig. 1. The line is a fit with a second-order polynomial model.

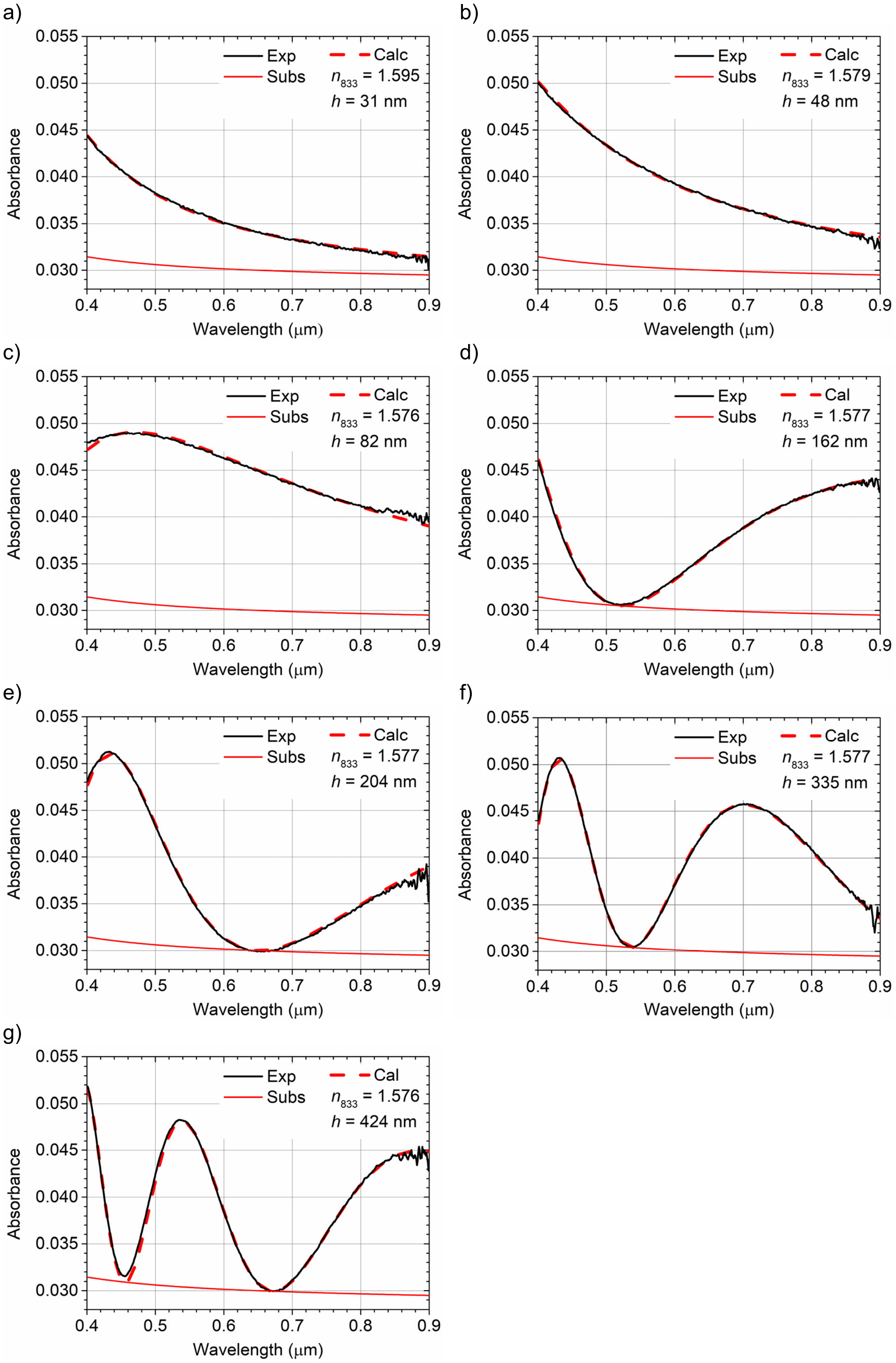

Figure S2. Experimental absorption spectra against air (black solid lines) and simulated interference patterns (red thick dashed lines) corresponding to PS films in Table 2 of the main text. The three verifications shown in Fig. 4 of the main text have been repeated here to better show the evolution of interferences with increasing thickness (**a**–**g**). The substrate spectrum (Eq. (6)) has also been included (red solid lines). Spectrum for wavelengths higher than 850 nm was discarded in most cases because of the increased noise level.

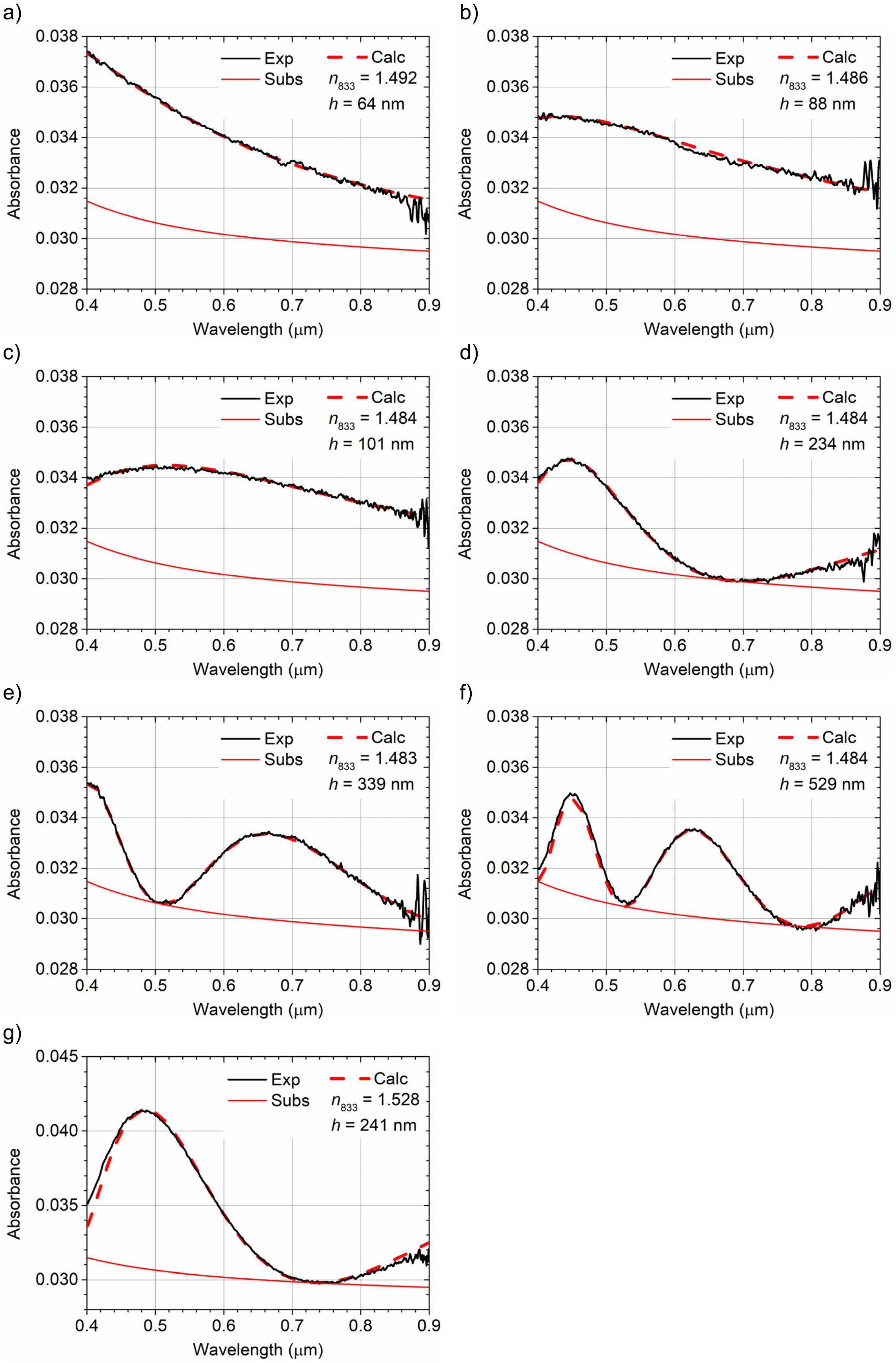

Figure S3. (a–g) Experimental absorption spectra against air (black solid lines) and simulated interference patterns (red thick dashed lines) corresponding to samples in Table 3 of the main text. g) The sample of gelatin presents a small absorption in short wavelengths leading to a decrease in transparency. In this case, the method has been applied in a window starting in 450 nm. Spectrum for wavelengths higher than 850 nm was discarded in most cases because of the increased noise level.

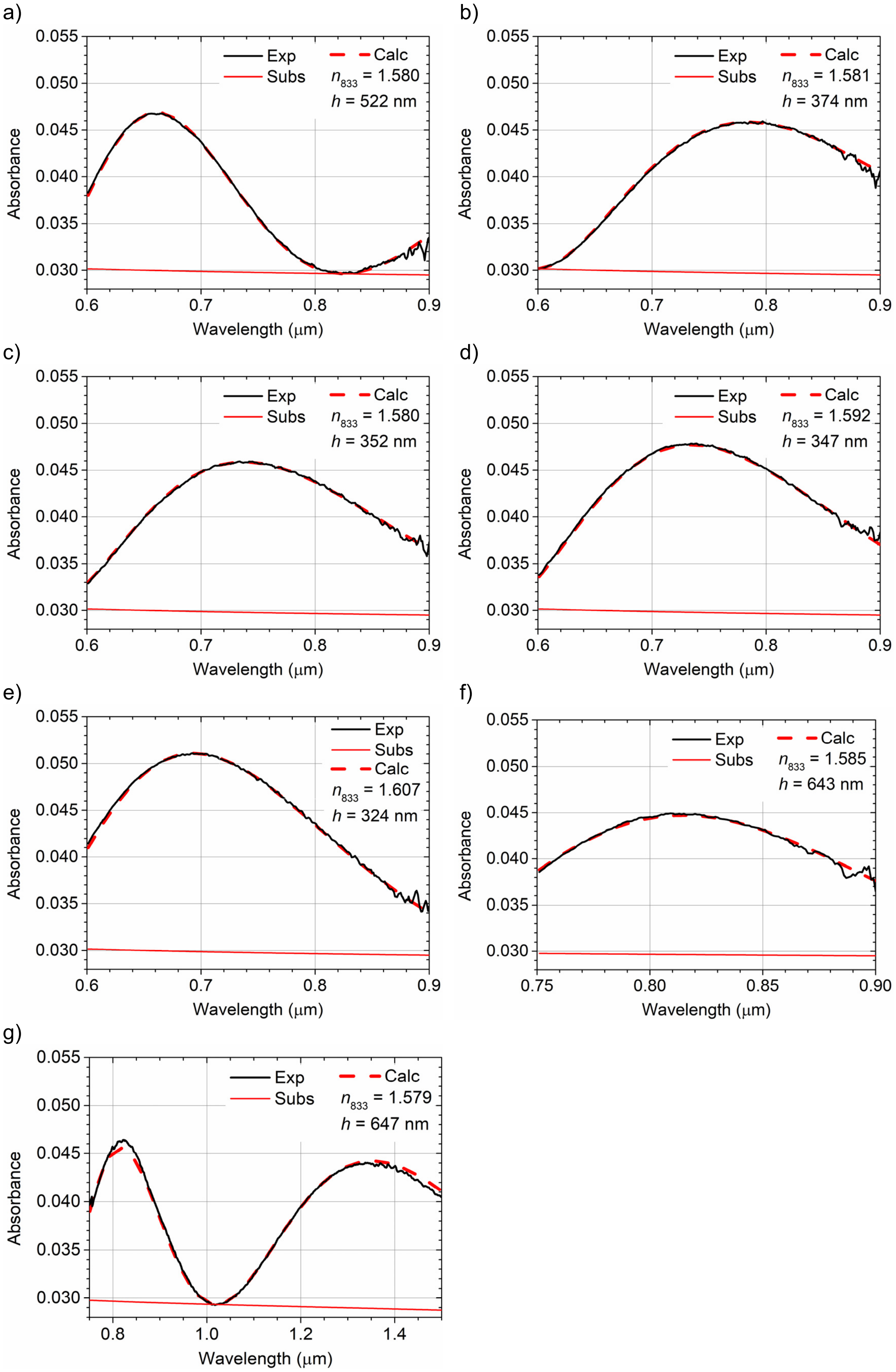

Figure S4. Experimental absorption spectra against air (black solid lines) and simulated interference patterns (red thick dashed lines) corresponding to doped PS films in Table 4 of the main text: (**a**) PDI-O 1%, (**b**), (**c**), (**d**) and (**e**) TPD 2.5%, 5%, 15% and 30%, respectively, and (**f**) FZ3 1%. Results for sample doped with FZ3 1% recorded with a V-670 UV-VIS-NIR spectrophotometer are shown in (**g**). The substrate spectrum (Eq. (8)) has also been included (red solid lines). Figures **b**) to **e**) show the increase in contrast of interferences due to an increase in the refractive index as a consequence of the increase in the concentration of TPD.
